# Uniformity and Deviation of Intra-axonal Cross-sectional Area Coverage of the Gray-to-White Matter Interface

**DOI:** 10.3389/fnins.2017.00729

**Published:** 2017-12-22

**Authors:** Stefan Sommer, Sebastian Kozerke, Erich Seifritz, Philipp Staempfli

**Affiliations:** ^1^Institute for Biomedical Engineering, University of Zurich and ETH Zurich, Zurich, Switzerland; ^2^MR-Center of the Departments of Psychiatry, Psychotherapy and Psychosomatics and of Child and Youth Psychiatry and Psychotherapy, Psychiatric Hospital, University of Zurich, Zurich, Switzerland; ^3^Department of Psychiatry, Psychotherapy and Psychosomatics, Hospital of Psychiatry, University of Zurich, Zurich, Switzerland

**Keywords:** tractography, fiber optimization, area coverage, intra-cellular cross-sectional area, gray-to-white matter interface, cortical folding

## Abstract

Diffusion magnetic resonance imaging (dMRI) is a compelling tool for investigating the structure and geometry of brain tissue based on indirect measurement of the diffusion anisotropy of water. Recent developments in global top-down tractogram optimizations enable the estimation of streamline weights, which characterize the connection between gray matter areas. In this work, the intra-axonal cross-sectional area coverage of the gray-to-white matter interface was examined by intersecting tractography streamlines with cortical regions of interest. The area coverage is the ratio of streamline weights divided by the surface area at the gray-to-white matter interface and assesses the estimated percentage which is covered by intra-axonal space. A high correlation (*r* = 0.935) between streamline weights and the cortical surface area was found across all regions of interest in all subjects. The variance across different cortical regions exhibits similarities to myelin maps. Additionally, we examined the effect of different diffusion gradient subsets at a lower, clinically feasible spatial resolution. Subsampling of the initial high-resolution diffusion dataset did not alter the tendency of the area coverage at the gray-to-white matter interface across cortical areas and subjects. However, single-shell acquisition schemes with lower *b*-values lead to a steady increase in area coverage in comparison to the full acquisition scheme at high resolution.

## Introduction

Diffusion tractography algorithms are able to reveal global fiber structures by estimating continuous streamline connections based on the local diffusion information throughout the brain (Jbabdi and Johansen-Berg, 2011; Jbabdi et al., 2015). In the last decade, the performance of tracking algorithms has significantly improved by considering the information contained in orientation distribution functions (ODF) or fiber orientation distribution (FOD), especially in regions with complex fiber configurations (Behrens et al., 2007; Fillard et al., 2011; Tournier et al., 2011). Two excellent reviews considering tractography pitfalls can be found in Jbabdi and Johansen-Berg (2011), Jones (2010), and Jones et al. (2012). Some of these issues are scanner related (e.g., eddy current induced distortions), caused by time restrictions, or due to the limited spatial resolution in clinically feasible acquisition protocols of diffusion data (about 2.5 mm isotropic), which is orders of magnitude larger than the diameter of a single axon. In the Human Connectome Project (HCP), data acquisition techniques and processing protocols have been significantly advanced (Glasser et al., 2013; Ugurbil et al., 2013). Besides progress in post-processing, the HCP provides high quality *in-vivo* diffusion data acquired with multi-shell diffusion gradient schemes and a high spatial resolution (Sotiropoulos et al., 2013). These datasets have been acquired on a dedicated MR scanner with optimized hardware and less severe acquisition time constraints in comparison to clinical applications.

However, not all problems can be addressed by improved hardware and acquisition protocols. One of the major remaining challenges is to reliably extract quantitative measures from tractograms across different populations.

Recent developments in global top-down tractography optimizations enable the estimation of fiber contributions and compartment fractions (Sherbondy et al., 2009, 2010; Smith et al., 2013, 2015b; Pestilli et al., 2014; Daducci et al., 2015). However, all of these optimization methods have their own pitfalls. An overview of problems and open challenges is given in Daducci et al. (2016).

Numerous models based on diffusion weighted imaging have been proposed to estimate parameters related to the restricted, intra-axonal compartment, commonly referred to as fiber density (Calamante et al., 2015; Raffelt et al., 2016). In the work of (Daducci et al., 2015; Smith et al., 2015b), an optimal weight for each streamline is determined according to a biologically motivated forward model and the measured diffusion signal. By assigning a weight of zero, false positive or implausible connections can be eliminated. The intra-axonal volume (i.e., fiber density) is calculated by multiplying each streamline contribution (fiber weight) by the streamline length. Therefore, the fiber weight is related to the intra-axonal cross-sectional area (Raffelt et al., 2012, 2016; Calamante et al., 2015; Smith et al., 2015b).

During development, the intricate folding of the cortex is formed in order to optimize the wiring and organization of the brain and fit a large cortex in a limited cranial volume (Fernández et al., 2016; Tallinen et al., 2016). The cortical surface area has been shown to be inversely correlated with gray matter thickness (Toro and Burnod, 2005; Pillay and Manger, 2007) and cortical areas seem to have evolved to optimize inter-areal connections by minimizing the required axonal volume within the white matter (Klyachko and Stevens, 2003). An increase of cortical thickness allows for more local axonal connections, whereby an increase of surface area might be needed to form more long-range axonal connections traversing the white matter. The intra-axonal cross-sectional area coverage is described by the ratio of streamline weights to gray-matter area and therefore indirectly assesses the estimated surface area percentage which is covered by intra-axonal space. Due to strict spatial constraints in the brain, we anticipate a homogeneous area coverage at the gray-to-white matter interface (G-WMI).

In this work, we tested this hypothesis by examining the tractography fiber weights from the COMMIT optimization and intersect the streamlines with cortical gray matter regions of interest (ROIs) to estimate the intra-axonal area coverage at the G-WMI in 10 healthy subjects from the HCP. Furthermore, the stability and replicability of these findings were tested for clinically feasible acquisition schemes by reducing the spatial resolution and utilizing only subsets of the diffusion gradient scheme of the HCP data. In addition, the area coverage was compared to myelin maps derived from anatomical *T*_1_ and *T*_2_–weighted anatomical images (Glasser and Van Essen, 2011).

## Materials and methods

### Human connectome datasets

MRI datasets of 10 healthy volunteers in the age range of 22–35 (6 female, 4 male) were obtained from the HCP Wu-Minn database (https://db.humanconnectome.org). Only non-restricted, anonymized open access datasets were used. Therefore, no ethical consent is necessary according to national laws and regulations. Subjects were scanned at a Siemens 3T scanner equipped with a dedicated, high performance gradient system capable of gradient strengths of 100 mT/m with special gradient amplifiers (Ugurbil et al., 2013). Three shells with *b*-values of 1,000, 2,000, and 3,000 s/mm^2^ were acquired with 90 diffusion encoding directions on each shell. The spatial resolution was 1.25 mm isotropic. Two phase-encoding direction reversed images for each diffusion direction were acquired. The non-diffusion weighted volumes with *b* = 0 were interleaved with DW volumes such that every sixteenth volume had no diffusion weighting. More details about the acquisition protocol can be found in Sotiropoulos et al. (2013).

The utilized diffusion datasets were already preprocessed by the HCP diffusion pipeline (Glasser et al., 2013). Briefly, distortions were corrected using a model-based approach that simultaneously takes into account susceptibility and eddy-current induced distortions, as well as head motion.

### Gradient non-linearity correction

The diffusion datasets in the HCP suffer from much stronger gradient non-linearities compared to datasets from conventional scanners. Therefore, the diffusion gradients are not spatially invariant across the field of view. As a consequence, the diffusion weighting can vary up to ±15% (Sotiropoulos et al., 2013) and should therefore not be neglected. Unfortunately, many software tools do not allow the use of spatially varying gradient tables within a single volume. In order to examine the severity and influence of the spatially varying diffusion weighting, we analyzed the effect of the gradient deviations for a simple tensor fit with and without accounting for the spatially varying b-matrix. The direction of the primary eigenvector of the tensor deviated up to 3° from the correctly processed tensor. Scaling of the diffusion weightings due to the varying b-factor emerged as the main effect of the gradient non-linearity. In order to obtain diffusion data with spatially constant *b*-values that conform to the limitations of the used software tools, we locally modeled the normalized diffusion signal with a mono-exponential signal decay for each shell. This leads to a corrected diffusion signal with a constant b-factor per volume and shell. The mono-exponential signal decay was modeled as follows:

(1) $$\begin{array}{r} \frac{s\left(\vec{x}\right)}{s_{0}}=e^{-b\left(\vec{x}\right)D} \end{array}$$

whereby $s\left(\vec{x}\right)$ denotes the local diffusion signal and *s*_0_ refers to the non-diffusion-weighted *b* = 0 image. $b\left(\vec{x}\right)$ is the diffusion b-factor and *D* represents the diffusion coefficient. With this approach, we assume a mono-exponential signal behavior for the local regime of ±15% of the *b*-value for each shell. With this approximation, the corrected diffusion signal *s*_*corr*_ can be calculated in each voxel with the given formula:

(2) $$\begin{array}{r} s_{corr}=s_{0}{\left(\frac{s_{act}\left(\vec{x}\right)}{s_{0}}\right)}^{\frac{b_{act}\left(\vec{x}\right)}{b_{corr}}} \end{array}$$

where $s_{act}\left(\vec{x}\right)$ represents the actual, measured diffusion signal at a given, spatially varying *b*-factor $b_{act}\left(\vec{x}\right)$ and *b*_*corr*_ represents the desired, corrected spatially constant *b*-factor.

### Tractography and global optimization

Constrained spherical deconvolution with recursive calibration of the response function (Tournier et al., 2007; Tax et al., 2014) with a maximal spherical harmonics order of 8 (Lmax = 8) and fiber tractography was performed in MRtrix3 (www.mrtrix.org) using the default iFOD2 probabilistic tractography algorithm (Tournier et al., 2010) with anatomical tissue priors (Smith et al., 2012). For the high-resolution dataset, the maximal fiber length was increased to 312.5 mm (250 times the voxel size). Additionally, the tractography seed points were determined dynamically using the SIFT model (Smith et al., 2015b). For all of the experiments, 5 million fibers were generated.

The top-down global tractography optimization was performed using the COMMIT framework (Daducci et al., 2015) by applying the Stick-Zeppelin-Ball model (Panagiotaki et al., 2012). The forward model consists of an intracellular stick model and an extracellular compartment modeled by a zeppelin to describe white matter (WM) and two distinct isotropic components for gray matter (GM) and cerebrospinal fluid (CSF) compartments.

The intracellular stick model was generated with a longitudinal diffusivity of *d*_∥_ = 1.7 × 10^−3^ mm^2^/s. In addition, in each voxel, a hindered contribution was included for every unique FOD peak using the Zeppelin model, assuming a perpendicular diffusivity of *d*_⊥_ = 0.5 × 10^−3^ mm^2^/s and a longitudinal diffusivity of *d*_∥_ = 1.7 × 10^−3^ mm^2^/s. Lastly, two isotropic compartments accounting for partial volume with GM and CSF were modeled with diffusivities of *d*∈ {1.7, 3.0} × 10^−3^ mm^2^/s. The normalized diffusion signal was multiplied by the voxel volume (in mm^3^) in order to ensure comparability across resolutions and with gray matter surface area. Stopping criteria for the convex optimization solver were set to either a maximum number of 500 iterations or a minimum relative change between two subsequent iterations of the objective function of 1 × 10^−4^.

We refer to fiber weights as the optimized contributions (${\tilde{x}}_{IC}$) of the intra-cellular stick model from the solution calculated by COMMIT.

### Fiber weights vs. gray matter parcellation

Each fiber contribution was projected onto GM regions by intersecting every streamline of the tractogram with cortical ROIs. Thereby, the weight of each streamline was assigned to the intersecting cortical ROIs extracted from the freesurfer parcellation, which is already part of the structural preprocessing of the HCP pipeline, and based on the Destrieux atlas (Destrieux et al., 2010). The parcellation scheme used during streamline intersection includes deep gray-matter structures. In the ideal case, each fiber contributes to exactly two ROIs. However, the node assignment is far from perfect (Daducci et al., 2016; Yeh et al., 2016). We tried to improve the mapping by allowing the extension of each fiber start-/endpoint in the direction of the first/last fiber segment for an additional stretch of 2 mm. This approach is more restrictive compared to the radial search proposed in Smith et al. (2015a) and further evaluated in Yeh et al. (2016). Nevertheless, we were still unable to perfectly map the tractogram onto the cortical parcellation. In order to measure the severity of the error emerging from the node assignment, we calculated and report the percentage of unassigned fiber weights to the total sum of fiber weights.

**Figure 1:**
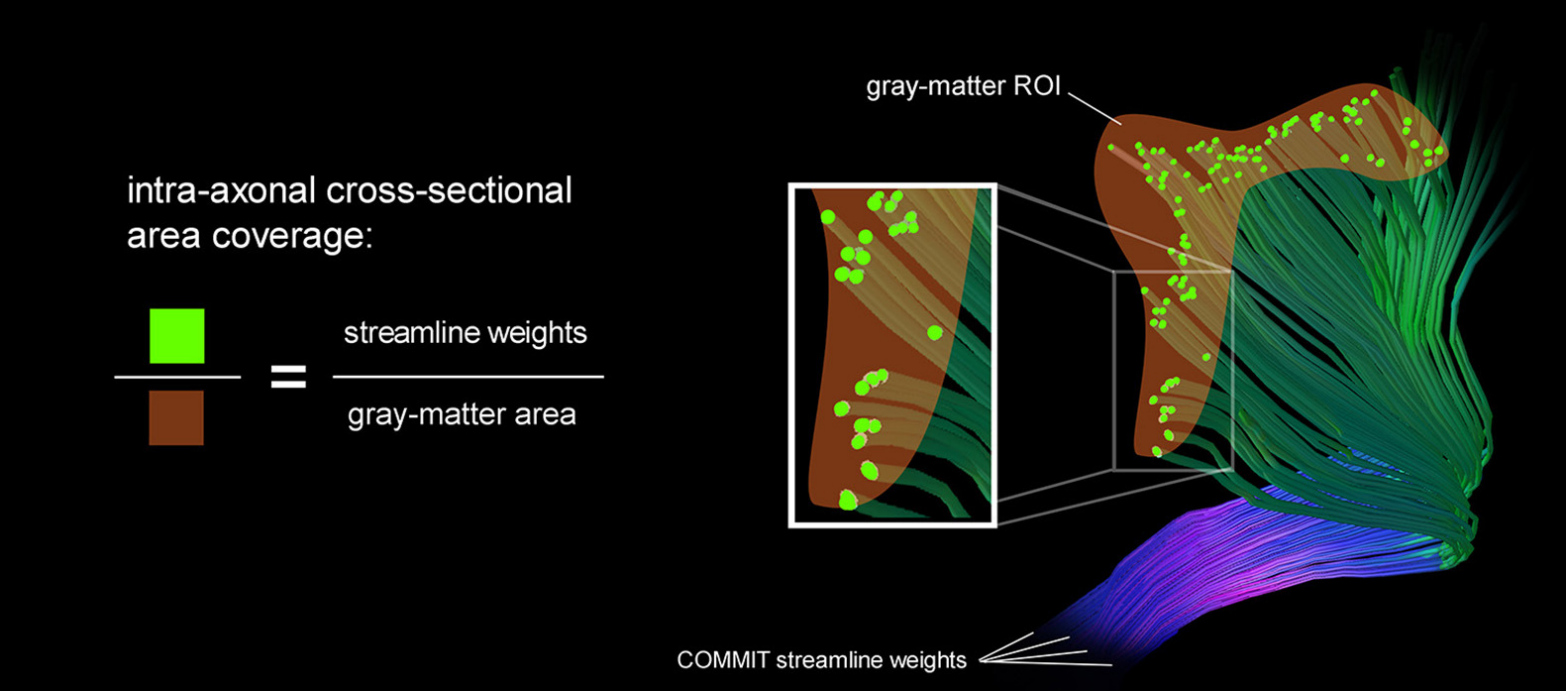
A schematic illustration of the area coverage of the G-WMI is shown. The streamline weights are interpreted as intra-axonal cross-sectional area.

We estimate the intra-axonal cross-sectional area of streamlines entering a gray matter area which is equivalent to the sum of streamline weights intersecting a particular gray-matter ROI. The area coverage is calculated as the sum of streamline weights divided by the surface area of the G-WMI. This fraction characterizes the percentage of surface area which is covered by axons entering or leaving the gray-matter. The intra-axonal area coverage of the G-WMI is therefore indirectly estimated by projecting the streamline weights resulting from the tractogram optimization onto the G-WMI. The derivation of the area coverage of the G-WMI is illustrated in Figure 1. Each COMMIT fiber weight describes the intra-axonal cross-sectional area represented by the particular streamline.

We calculated the Pearson correlation between gray matter surface area and streamline weights across all subjects. Furthermore, a permutation test was applied to test for statistical significance by randomly interchanging the assignment of GM surface areas to streamline weights. A total number of one million random permutations was performed.

### Replicability for clinically feasible sequences

We examined the intra-axonal area coverage at the G-WMI in high-quality datasets from the human connectome scanner. In order to test replicability in a clinically feasible setting, the high-resolution multi-shell diffusion acquisitions were spatially down-sampled by a factor of two to a voxel resolution of 2.5 mm isotropic using sinc-interpolation. Additionally, the gradient scheme was under-sampled to single-shell acquisition schemes (for *b* = 1,000, *b* = 2,000 and *b* = 3,000 s/mm^2^) with gradient subsets of 45 and 64 directions (for each shell). The minimum number of 45 directions was chosen to sufficiently characterize the diffusion signal (Tournier et al., 2013). The gradient subsets for each shell were selected with respect to the minimal energy according to the electrostatic repulsion of the sampling points. However, it is important to mention that a subset of a predefined sampling scheme will not sample the sphere as uniformly as an optimal scheme in a separate acquisition. Additionally, the full gradient scheme was also evaluated and utilized as reference. The complete processing pipeline starting from the constrained spherical deconvolution, tractography, and COMMIT optimization was performed separately for each gradient sampling scheme. The area coverage at the G-WMI was determined for each dataset as described in section Fiber Weights vs. Gray Matter Parcellation.

Finally, the root mean square error (RMSE) of the area coverage at the G-WMI between the high and low-resolution datasets and the different gradient subsets was calculated per subject.

### Myelin maps

The human-connectome structural pre-processing pipeline does provide myelin maps derived from the anatomical *T*_1_ and *T*_2_–weighted images (Glasser and Van Essen, 2011; Glasser et al., 2013). We extracted the myelin maps from the same subjects and calculated a group averaged myelin map based on the same parcellation scheme. Furthermore, we calculated the correlation between the myelin map and the area coverage for all subjects and the group average.

## Results

### Fiber weights and cortical parcellation

**Figure 2:**
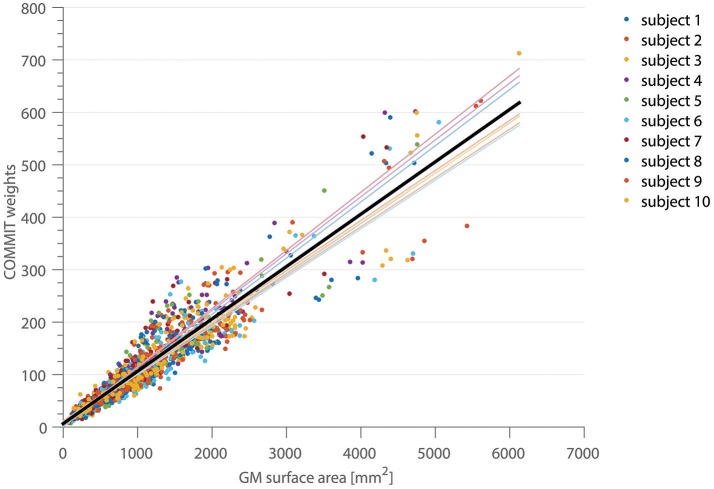
The correlation between the COMMIT weights and the GM surface area is shown for 10 subjects in 150 ROIs of the Destrieux atlas. The thin colored lines represent the linear regression for each subject, the solid black line depicts the regression across all subjects.

In Figure 2, the correlation between the COMMIT weights and the cortical ROI surface area for the high-resolution, full gradient scheme is shown. Each color depicts a separate subject. The mean area coverage resulted in 11.01 ± 2.57% of the G-WMI (see also Table 1). The Pearson correlation between COMMIT weights and GM surface is 0.935. The *p*-value of 10^−6^ obtained by one million random permutations of the ROI areas demonstrates a statistically significant correlation.

**Table 1:** Mean intra-axonal area coverage and standard deviation of the G-WMI and percentage of unassigned fiber weights are listed for different resolutions and gradient sub-sets.

**Resolution (mm) isotropic**	**Gradient set**	**Mean and standard deviation of area coverage (%)**	**Unassigned fiber weights (%)**
1.25	Multi-shell, full	11.01 ± 2.57	21.41 ± 1.47
2.5	Multi-shell, full	13.41 ± 2.91	17.61 ± 1.11
2.5	*b* = 1,000, 45 dirs	19.77 ± 3.93	14.40 ± 1.21
2.5	*b* = 2,000, 45 dirs	15.28 ± 3.23	16.06 ± 1.26
2.5	*b* = 3,000, 45 dirs	13.45 ± 3.01	17.37 ± 1.26
2.5	*b* = 1,000, 64 dirs	19.09 ± 3.87	14.67 ± 1.12
2.5	*b* = 2,000, 64 dirs	15.04 ± 3.29	16.19 ± 1.21
2.5	*b* = 3,000, 64 dirs	13.11 ± 2.96	17.52 ± 1.18

**Figure 3:**
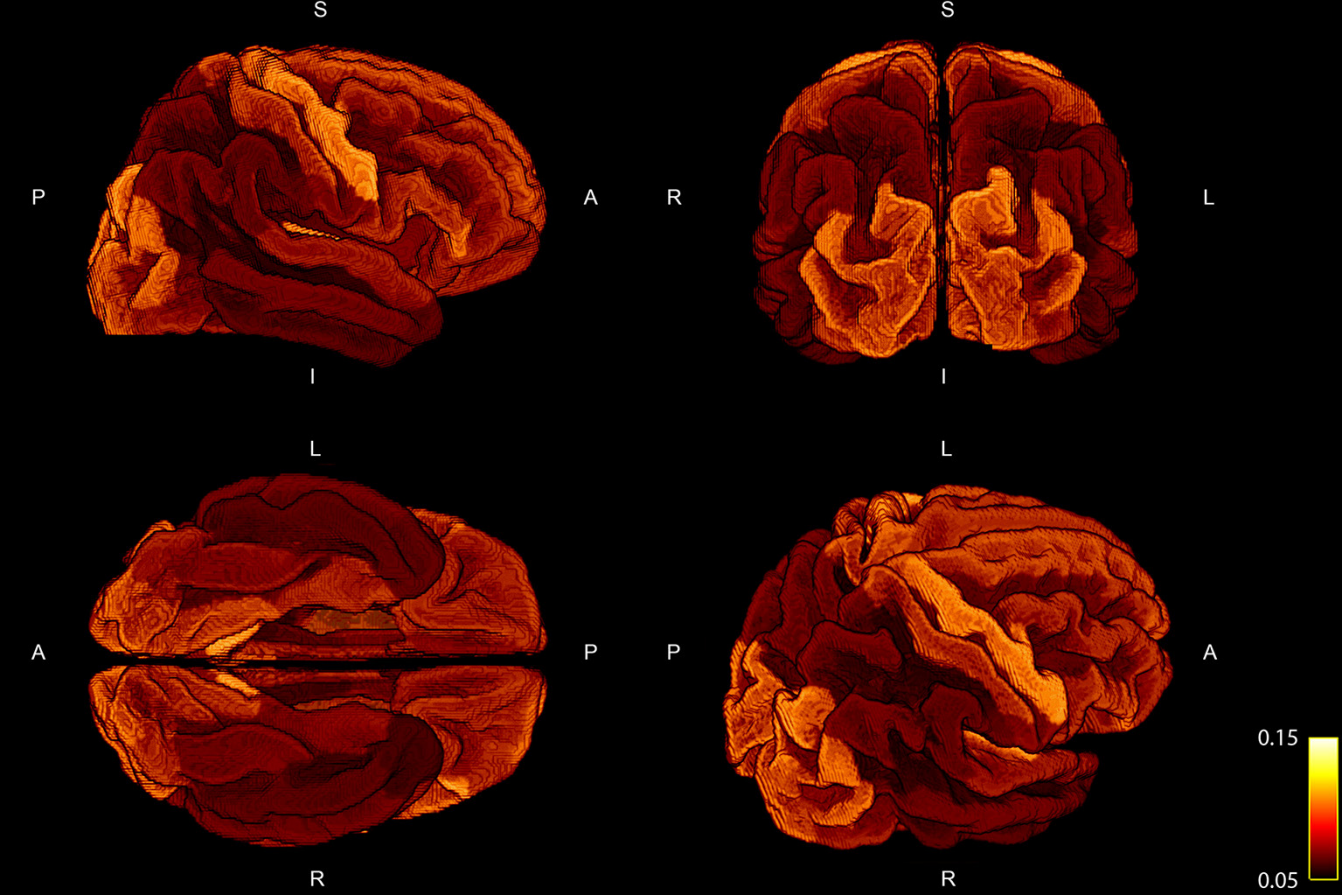
Four different views of the group mean ratio between the COMMIT weights and the ROI areas. Group mean ratios are projected onto the standardized cortical parcellation.

The percentage of fiber weights which was not taken into account due to the fact that the corresponding streamline could not be assigned to any of the cortical ROIs resulted in 21.41 ± 1.47%. In order to examine the deviation of the regression, we show the mean ratio across the 10 subject for each ROI projected onto the standardized brain parcellation in Figure 3, where four different views of the cortical regions are depicted. Besides a very high symmetry between the left and right hemisphere, the notable regions with an increased intra-axonal area coverage are the primary motor, visual, and auditory regions. The primary sensory cortex is also slightly elevated. A decrease in area coverage of the G-WMI can be observed in e.g., the temporal poles.

### Subsampling of the gradient scheme

**Figure 4:**
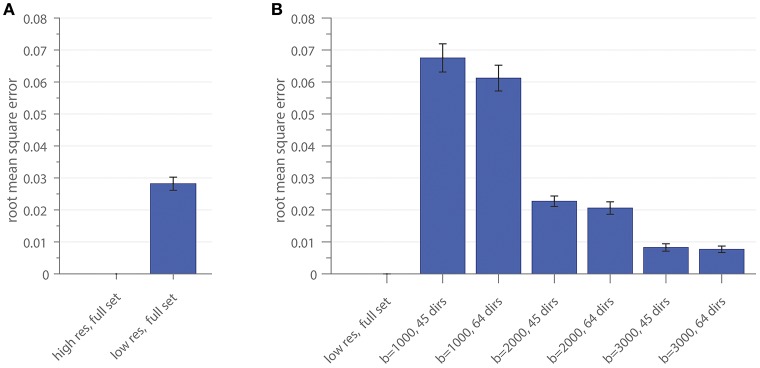
The root mean square error of the area coverage at the G-WMI is shown for the down-sampled spatial resolution **(A)** using the high-resolution as a reference and **(B)** the subsampled gradient schemes with the low-resolution full set as reference.

The RMSE of the ratio for different resolutions and gradient subsets is shown in Figure 4. Figure 4A depicts the RMSE of the low-resolution dataset, including all diffusion directions (low res, full set), in comparison to the result of the high-resolution dataset, including all diffusion directions (high res, full set), as a reference. In Figure 4B, the RMSE of the gradient subsampling schemes for the different shells is depicted with the low-resolution full gradient set as reference. The error-bars in both subplots show the standard deviation across the 10 subjects. The error can be interpreted as the deviation of the surface area which is covered by the cross-sectional intra-axonal surface area in comparison to the respective full set (reference).

**Figure 5:**
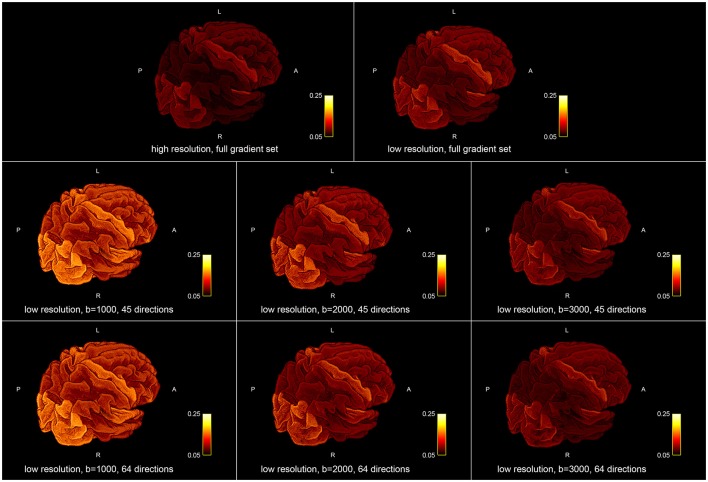
Absolute intra-axonal area coverage at the G-WMI for different resolutions **(Top)** and gradient sub sets **(Bottom)**.

**Figure 6:**
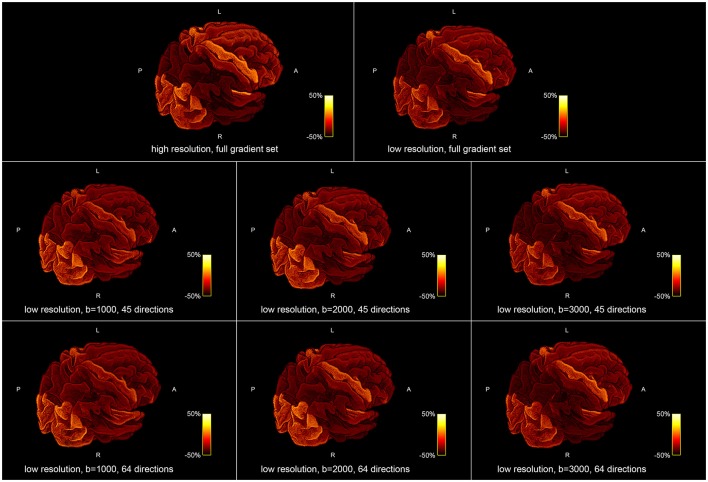
Percentage deviation of the mean area coverage at the G-WMI for different resolutions **(Top)** and gradient sub sets **(Bottom)**.

Figure 5 shows the influence of down-sampling the resolution and of subsampling the gradient set with respect to variations of the absolute intra-axonal area coverage of the G-WMI across the brain. The identical coloring scheme was used for each subplot. The gradient subsets with lower *b*-values tend to overestimate the area coverage in comparison to the high *b*-value and multi-shell acquisition. The normalized area coverage (percentage deviation of the mean) is shown in Figure 6. All subsets show similar deviations (increase and decrease) from the mean area coverage in the same anatomical regions.

In Table 1, we report the intra-axonal area coverage of the G-WMI as percentage coverage of the intra-axonal cross-sectional area of the total gray matter surface area at the G-WMI for the different resolutions and gradient subsets (range 11.01–19.77%). The standard deviation of the area coverage describes the variance across different ROIs. In comparison, the deviation of the averaged intra-axonal cross-sectional area coverage across subjects for a particular ROI is much smaller. Additionally, we also report the percentage of unassigned fiber weights in order to estimate errors from the node-assignment (range 14.40–21.41%).

**Figure 7:**
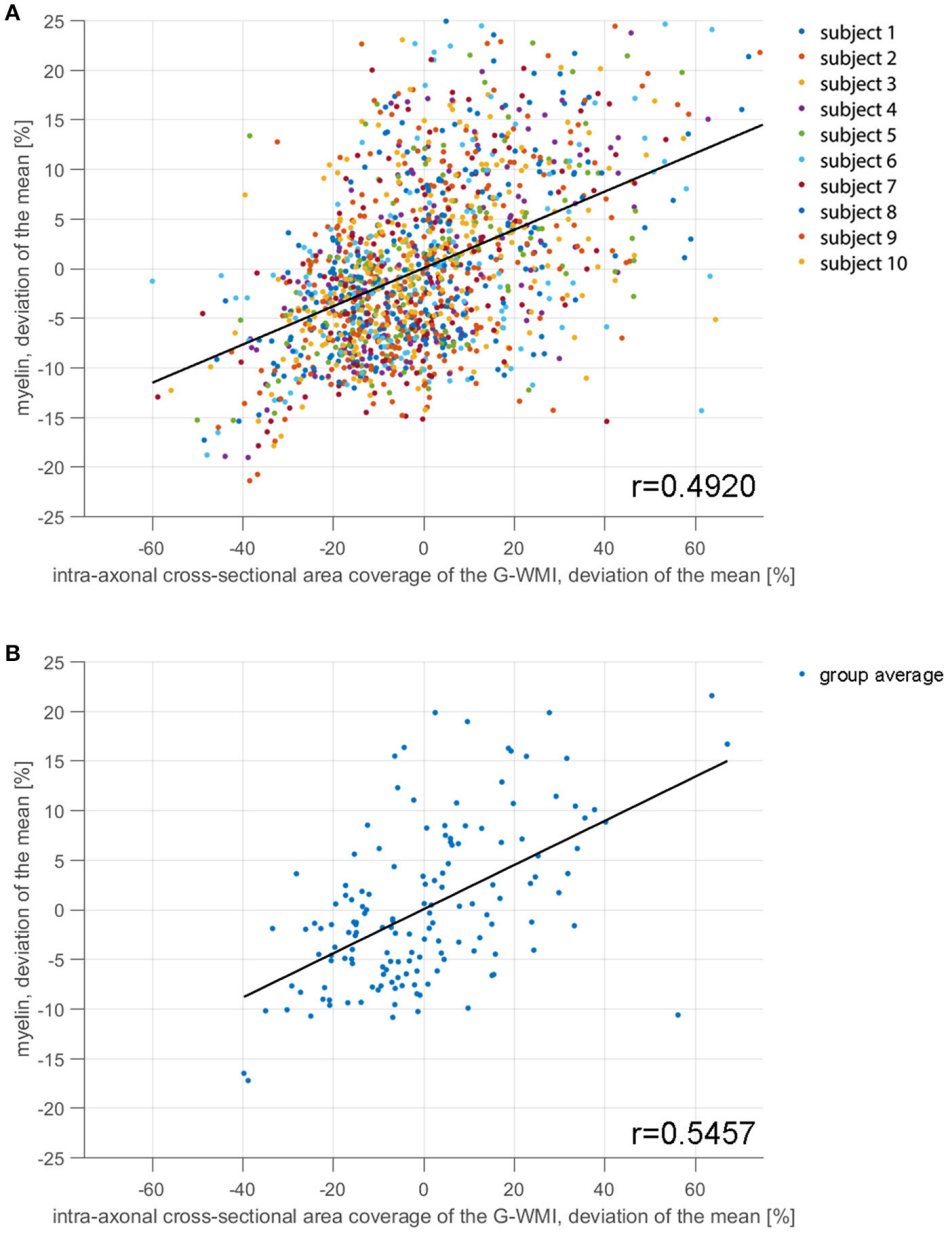
The correlation between area coverage and myelin is shown for 10 subjects in 150 ROIs of the Destrieux atlas in subplot **(A)**. Subplot **(B)** shows the correlation averaged across the 10 subjects for each ROI.

In Figure 7, we present a scattering plot comparing the area coverage to myelin maps provided by the human connectome structural pre-processing pipeline. Figure 7A shows all subjects and ROIs with a moderate correlation of *r* = 0.4920. Figure 7B shows the group averaged comparison with a slightly higher correlation of *r* = 0.5457. The range of deviation of the mean (−50%, 50%) is higher for the area coverage in comparison to the myelin percentage deviation (−18%, 18%).

**Figure 8:**
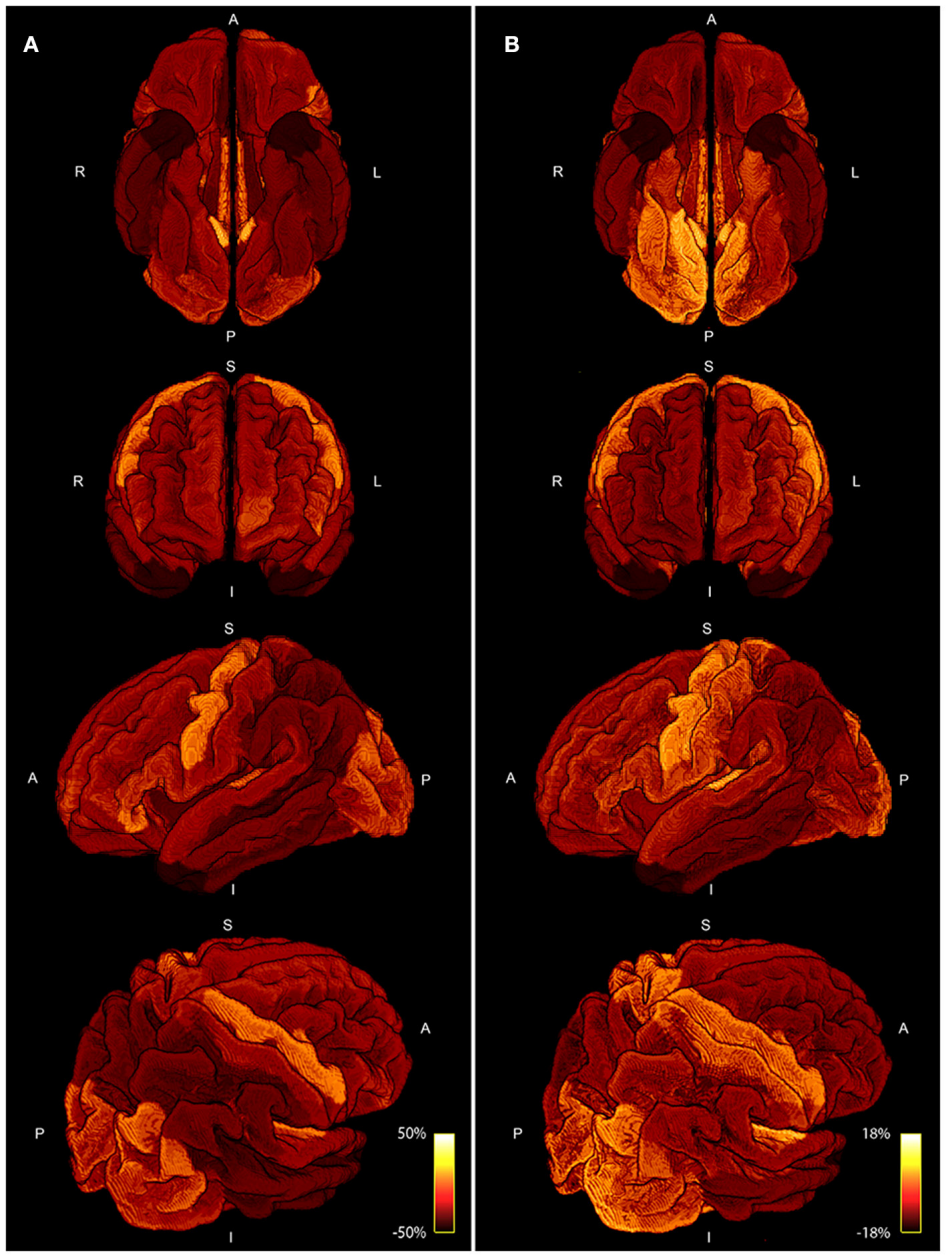
Four different views of the area coverage **(A)** are compared to the myelin map projected onto the standardized cortical parcellation **(B)**. Both modalities are group averaged and the percentage deviation of the mean is shown to visualize spatial variations. The range of area coverage in **(A)** is larger (−50%, 50%) in comparison to the variation in of myelin in **(B)** (−18%, 18%).

Figure 8 shows a comparison of the area coverage (Figure 8A) with the averaged myelin maps (Figure 8B) provided by the human connectome structural pre-processing pipeline from the 10 subjects. The same parcellation scheme was applied. The percentage deviation of the mean is shown to illustrate deviations in the area coverage and myelination.

## Discussion

In this work, we tested the hypothesis of a homogeneous intra-axonal cross-sectional area coverage of the G-WMI by correlating tractography fiber weights from the COMMIT optimization with the gray matter surface area at the interface. Analysis was performed on the high-resolution datasets of 10 healthy subjects from the HCP using the full multi-shell diffusion gradient sampling scheme. We found a high correlation (*p* = 0.935) between the intra-axonal cross-sectional area (represented by the fiber weights) and the gray matter surface area across different ROIs and subjects. However, we also noted consistent deviations in various structures across all subjects.

The positive correlations indicate that, as expected, a larger ROI might intersect with more streamlines than a smaller ROI. Nevertheless, the magnitude of the correlation and the accordance of the ratio of fiber-weights to ROI area across all 10 subjects is remarkable. During the COMMIT optimization, the intra-axonal volume is calculated by multiplying the fiber-weights with the fiber segment length. Hence, dividing the fiber volume by the fiber length, results in a cross-sectional area (the fiber weight). It is thus tempting to directly interpret the fiber-weights as a sum of intra-cellular cross-sectional areas of axons, represented by a particular streamline. However, it is important to keep in mind that we assign the MR visible signal to a voxel volume, which is only an approximation (e.g., myelin is ignored completely). Regardless, following this assumption, the ratio of fiber-weights to GM area represents the intra-cellular cross-sectional area coverage of the G-WMI. The variance of this ratio among different cortical ROIs is visualized in Figures 3, 5, 6, and the standard deviation across ROIs is listed in Table 1. Besides a high symmetry of the left and right hemispheres, the primary visual, motor and auditory areas exhibit an increased density at the G-WMI. The communality between these regions is that they all directly process external “inputs” (and also “outputs” in case of the primary motor cortex).

The comparison of area coverage to myelin in Figure 8 reveals a similar pattern of increased (or decreased) areas of the brain. Slight asymmetries between hemispheres are consistent across both modalities, e.g., an increase in the left frontal cortex in comparison to the right side. The primary motor, visual, and auditory cortices are elevated in both modalities, whereas the temporal poles are lowered in both measures. Most notable differences besides scaling are revealed in the axial view in Figure 8, especially in the inferior posterior part of the brain. The primary sensory cortex is also more prominent in myelin. A resemblance between intra-axonal area coverage and myelin is partly explicable—a local increase in axonal connections would also lead to an increase in myelin if the degree of myelination is constant. However, myelination might also vary independent of the axonal density. Furthermore, the spatially varying distribution of axonal diameter and myelin thickness (g-ratio) will also influence the direct relation between axonal cross-sectional area and myelination. These factors might also explain the only moderate correlation of around 0.5 presented in Figure 7. Nevertheless, the coherence between high and low area coverage/myelination is even more convincing by considering that both measures are extracted from different modalities and processing steps.

The reproducibility of our findings was also tested for clinically feasible acquisition schemes. We therefore artificially reduced the spatial resolution and subsampled the diffusion gradient scheme to single-shell subsets and different b-factors with different number of gradient directions.

Similar intra-axonal area coverage distributions across the complete G-WMI were found in all subsampled datasets. Nevertheless, especially for lower *b*-values, the absolute area coverage was over-estimated in comparison to the full diffusion gradient scheme, whereas distributions based on high *b*-value (b = 3000) sets with 45 or 64 directions were almost identical to the full multi-shell dataset.

The use of larger ROIs may cancel out local variations in the tractogram due to spatial averaging. Hence, a more finely grained parcellation scheme could potentially reveal further differences between the different gradient schemes.

Additionally, the high-resolution dataset exhibited a slightly decreased area coverage in comparison to the down-sampled low-resolution dataset, however, this might also be caused by the increased number of unassigned fiber weights during the node-assignment.

We found a conforming intra-axonal cross-sectional area coverage at the G-WMI across different areas and subjects, however there were also marked variations in area coverage in some cortical areas. Whilst this effect is stable across subjects and across multiple gradient schemes with varying *b*-values and spatial resolutions, it is difficult to determine the cause of these fluctuations. The deviation from the mean area coverage could be caused by biological differences or intrinsic properties of the processing pipeline (e.g., choice of tractography algorithm).

However, the comparison to myelin reveals a similar spatial pattern, even though the two measures are derived from different acquisitions and methodologies. Therefore, it is less plausible that these fluctuations are caused by the applied diffusion processing pipeline.

Additionally, the evaluation of cortical thickness and surface area based on anatomical scans is an approximation e.g., due to limited resolution and might also suffer from artifacts and inaccuracies (Zilles and Amunts, 2015).

Furthermore, the node-assignment remains an issue and might also have influenced results. If we compare the percentage of unassigned fiber-weights, a higher spatial resolution and improved angular resolution due to higher *b*-values or more diffusion directions negatively impact the node-assignment. We chose to use a more restrictive method to prevent incorrect assignments of streamlines to ROIs. A different strategy would be to remove unassigned streamlines prior to the COMMIT optimization, although this might lead to missing atoms in the dictionary (Daducci et al., 2016). However, as presented in Yeh et al. (2017), the mesh-based anatomically-constrained tractography where unified tissue priors are used for tractography and parcellation will be better than any heuristic node assignment strategy.

Unfortunately, the beneficial effects of improved tractograms (at higher spatial and angular resolution) might be mitigated in the applied analysis. Apart from the need of an accurate and reliable assignment of streamlines to GM ROIs, it is also crucial to observe and minimize problems and pitfalls during the optimization as discussed in Daducci et al. (2016) and Sommer et al. (2017). Additionally, it is still unclear if the fully sampled multi-shell acquisition scheme is ideal for the fitting of the utilized microstructure model for the global tractography optimization or if each q-space sample should be weighted according to e.g., the signal-to-noise ratio or the number of sampling points during optimization.

Regardless, a striking resemblance is observed between the axon packing density at the G-WMI and intra-cortical myelin maps derived from T_1_, T_2_, and proton-density weighted images (Glasser and Van Essen, 2011; Rowley et al., 2015).

In conclusion, we presented a novel method that allows the indirect quantification of the axonal packing density at the G-WMI, based on fiber weights derived from tractography optimization. Furthermore, the hypothesis that the intra-axonal cross-sectional area is proportional to the cortical surface area is supported by the presented experiments and can be replicated with clinically feasible spatial resolutions, even with a single shell acquisition scheme.

## Ethics statement

Publicly available data (from the human connectome project) was used in this study.

## Author contributions

SS, PS, SK, ES: Conceived and designed the experiments; SS: Performed the experiments and analyzed the data; SS, PS: Contributed reagents, materials, analysis tools; SS, PS, SK, ES: Wrote the paper.

### Conflict of interest statement

The authors declare that the research was conducted in the absence of any commercial or financial relationships that could be construed as a potential conflict of interest.

## References

[B1] Behrens T. E. Berg H. J. Jbabdi S. Rushworth M. F. Woolrich M. W. (2007). Probabilistic diffusion tractography with multiple fibre orientations: what can we gain? Neuroimage 34, 144–155. 10.1016/j.neuroimage.2006.09.018 17070705 PMC7116582

[B2] Calamante F. Smith R. E. Tournier J.-D. Raffelt D. Connelly A. (2015). Quantification of voxel-wise total fibre density: investigating the problems associated with track-count mapping. Neuroimage 117, 284–293. 10.1016/j.neuroimage.2015.05.070 26037054

[B3] Daducci A. Dal Palu A. Descoteaux M. Thiran J.-P. (2016). Microstructure informed tractography: pitfalls and open challenges Microstructure informed tractography: pitfalls and open challenges. Front. Neurosci. 10:247. 10.3389/fnins.2016.00247 27375412 PMC4893481

[B4] Daducci A. Dal Palu A. Lemkaddem A. Thiran J.-P. (2015). COMMIT: Convex Optimization Modeling for Microstructure Informed Tractography. IEEE Trans. Med. Imaging 34, 246–257. 10.1109/TMI.2014.2352414 25167548

[B5] Destrieux C. Fischl B. Dale A. Halgren E. (2010). Automatic parcellation of human cortical gyri and sulci using standard anatomical nomenclature. Neuroimage 53, 1–15. 10.1016/j.neuroimage.2010.06.010 20547229 PMC2937159

[B6] Fernández V. Llinares-Benadero C. Borrell V. (2016). Cerebral cortex expansion and folding: what have we learned? EMBO J. 35, 1021–1044. 10.15252/embj.201593701 27056680 PMC4868950

[B7] Fillard P. Descoteaux M. Goh A. Gouttard S. Jeurissen B. Malcolm J.. (2011). Quantitative evaluation of 10 tractography algorithms on a realistic diffusion MR phantom. Neuroimage 56, 220–234. 10.1016/j.neuroimage.2011.01.032 21256221

[B8] Glasser M. F. Van Essen D. C. (2011). Mapping human cortical areas *in vivo* based on myelin content as revealed by T1- and T2-weighted MRI. J. Neurosci. 31, 11597–11616. 10.1523/JNEUROSCI.2180-11.2011 21832190 PMC3167149

[B9] Glasser M. F. Sotiropoulos S. N. Wilson J. A. Coalson T. S. Fischl B. Andersson J. L.. (2013). The minimal preprocessing pipelines for the Human Connectome Project. Neuroimage 80, 105–124. 10.1016/j.neuroimage.2013.04.127 23668970 PMC3720813

[B10] Jbabdi S. Johansen-Berg H. (2011). Tractography: where do we go from here? Brain Connect. 1, 169–183. 10.1089/brain.2011.0033 22433046 PMC3677805

[B11] Jbabdi S. Sotiropoulos S. N. Haber S. N. Van Essen D. C. Behrens T. E. (2015). Measuring macroscopic brain connections *in vivo*. Nat. Neurosci. 18, 1546–1555. 10.1038/nn.4134 26505566

[B12] Jones D. K. (2010). Challenges and limitations of quantifying brain connectivity *in vivo* with diffusion MRI. Imaging Med. 2, 341–355. 10.2217/iim.10.21

[B13] Jones D. K. Knösche T. R. Turner R. (2012). White matter integrity, fiber count, and other fallacies: the do's and don'ts of diffusion MRI. Neuroimage 73, 239–254. 10.1016/j.neuroimage.2012.06.081 22846632

[B14] Klyachko V. A. Stevens C. F. (2003). Connectivity optimization and the positioning of cortical areas. Proc. Natl. Acad. Sci. U.S.A. 100, 7937–7941. 10.1073/pnas.0932745100 12796510 PMC164691

[B15] Panagiotaki E. Schneider T. Siow B. Hall M. G. Lythgoe M. F. Alexander D. C. (2012). Compartment models of the diffusion MR signal in brain white matter: a taxonomy and comparison. Neuroimage 59, 2241–2254. 10.1016/j.neuroimage.2011.09.081 22001791

[B16] Pestilli F. Yeatman J. D. Rokem A. Kay K. N. Wandell B. A. (2014). Evaluation and statistical inference for human connectomes. Nat. Methods 11, 1058–1063. 10.1038/nmeth.3098 25194848 PMC4180802

[B17] Pillay P. Manger P. R. (2007). Order-specific quantitative patterns of cortical gyrification. Eur. J. Neurosci. 25, 2705–2712. 10.1111/j.1460-9568.2007.05524.x 17459107

[B18] Raffelt D. A. Tournier J.-D. Smith R. E. Vaughan D. N. Jackson G. Ridgway G. R.. (2016). Investigating white matter fibre density and morphology using fixel-based analysis. Neuroimage 144(Pt A), 58–73. 10.1016/j.neuroimage.2016.09.029 27639350 PMC5182031

[B19] Raffelt D. Tournier J.-D. Rose S. Ridgway G. R. Henderson R. Crozier S.. (2012). Apparent Fibre Density: a novel measure for the analysis of diffusion-weighted magnetic resonance images. Neuroimage 59, 3976–3994. 10.1016/j.neuroimage.2011.10.045 22036682

[B20] Rowley C. D. Bazin P.-L. Tardif C. L. Sehmbi M. Hashim E. Zaharieva N.. (2015). Assessing intracortical myelin in the living human brain using myelinated cortical thickness. Front. Neurosci. 9:396. 10.3389/fnins.2015.00396 26557052 PMC4615825

[B21] Sherbondy A. J. Dougherty R. F. Ananthanarayanan R. Modha D. S. Wandell B. A. (2009). Think global, act local; projectome estimation with BlueMatter. Med. Image Comput. Comput. Assist. Interv. 12, 861–868. 10.1007/978-3-642-04268-3_106 20426069 PMC3076280

[B22] Sherbondy A. J. Rowe M. C. Alexander D. C. (2010). MicroTrack: an algorithm for concurrent projectome and microstructure estimation. Med. Image Comput. Comput. Assist. Interv. 13, 183–190. 10.1007/978-3-642-15705-9_23 20879230

[B23] Smith R. E. Tournier J. D. Calamante F. Connelly A. (2012). Anatomically-constrained tractography: improved diffusion MRI streamlines tractography through effective use of anatomical information. Neuroimage 62, 1924–1938. 10.1016/j.neuroimage.2012.06.005 22705374

[B24] Smith R. E. Tournier J. D. Calamante F. Connelly A. (2015a). The effects of SIFT on the reproducibility and biological accuracy of the structural connectome. Neuroimage 104, 253–265. 10.1016/j.neuroimage.2014.10.004 25312774

[B25] Smith R. E. Tournier J.-D. Calamante F. Connelly A. (2013). SIFT: Spherical-deconvolution informed filtering of tractograms. Neuroimage 67, 298–312. 10.1016/j.neuroimage.2012.11.049 23238430

[B26] Smith R. E. Tournier J.-D. Calamante F. Connelly A. (2015b). SIFT2: enabling dense quantitative assessment of brain white matter connectivity using streamlines tractography. Neuroimage 119, 338–351. 10.1016/j.neuroimage.2015.06.092 26163802

[B27] Sommer S. Kozerke S. Seifritz E. Staempfli P. (2017). Fiber up-sampling and quality assessment of tractograms - towards quantitative brain connectivity. Brain Behav. 7:e00588. 10.1002/brb3.588 28127510 PMC5256175

[B28] Sotiropoulos S. N. Jbabdi S. Xu J. Andersson J. L. Moeller S. Auerbach E. J.. (2013). Advances in diffusion MRI acquisition and processing in the Human Connectome Project. Neuroimage 80, 125–143. 10.1016/j.neuroimage.2013.05.057 23702418 PMC3720790

[B29] Tallinen T. Chung J. Y. Rousseau F. Girard N. Lefèvre J. Mahadevan L. (2016). On the growth and form of cortical convolutions. Nat. Phys. 12, 88–593. 10.1038/nphys3632

[B30] Tax C. M. W. Jeurissen B. Vos S. B. Viergever M. A. Leemans A. (2014). Recursive calibration of the fiber response function for spherical deconvolution of diffusion MRI data. Neuroimage 86, 67–80. 10.1016/j.neuroimage.2013.07.067 23927905

[B31] Toro R. Burnod Y. (2005). A morphogenetic model for the development of cortical convolutions. Cereb. Cortex 15, 1900–1913. 10.1093/cercor/bhi068 15758198

[B32] Tournier J. D. Calamante F. Connelly A. (2013). Determination of the appropriate b value and number of gradient directions for high-angular-resolution diffusion-weighted imaging. NMR Biomed. 26, 1775–1786. 10.1002/nbm.3017 24038308

[B33] Tournier J.-D. Calamante F. Connelly A. (2007). Robust determination of the fibre orientation distribution in diffusion MRI: non-negativity constrained super-resolved spherical deconvolution. Neuroimage 35, 1459–1472. 10.1016/j.neuroimage.2007.02.016 17379540

[B34] Tournier J.-D. Calamante F. Connelly A. (2010). Improved probabilistic streamlines tractography by 2nd order integration over fibre orientation distributions, in Conference: 18th International Society of Magnetic Resonance in Medicine (Stockholm). ISMRM, 1670.

[B35] Tournier J.-D. Mori S. Leemans A. (2011). Diffusion tensor imaging and beyond. Magn. Reson. Med. 65, 1532–1556. 10.1002/mrm.22924 21469191 PMC3366862

[B36] Ugurbil K. Xu J. Auerbach E. J. Moeller S. Vu A. T. Duarte-Carvajalino J. M.. (2013). Pushing spatial and temporal resolution for functional and diffusion MRI in the Human Connectome Project. Neuroimage 80, 80–104. 10.1016/j.neuroimage.2013.05.012 23702417 PMC3740184

[B37] Yeh C. Smith R. E. Dhollander T. Connelly A. (2017). Mesh-based anatomically-constrained tractography for effective tracking termination and structural connectome construction, in Conference: 25th International Society of Magnetic Resonance in Medicine (Honolulu, HI). ISMRM, 58.

[B38] Yeh C.-H. Smith R. E. Dhollander T. Calamante F. Connelly A. (2016). The influence of node assignment strategies and track termination criteria on diffusion MRI-based structural connectomics, in Conference: 24th International Society of Magnetic Resonance in Medicine (Singapore). ISMRM, 118.

[B39] Zilles K. Amunts K. (2015). Anatomical Basis for Functional Specialization, in fMRI: From Nuclear Spins to Brain Functions, eds Uludag K. Ugurbil K. Berliner L. (New York, NY: Spinger), 27–66.

